# Advances in Laboratory Diagnosis

**DOI:** 10.3390/diagnostics16040580

**Published:** 2026-02-14

**Authors:** Giuseppe Lippi, Camilla Mattiuzzi

**Affiliations:** 1 Section of Clinical Biochemistry, University of Verona, Piazzale Ludovico Antonio Scuro 10, 37134 Verona, Italy; 2 Medical Direction, Rovereto Hospital, Privincial Trust for Social and Sanitary Services, Corso Verona 4, 38068 Rovereto, Italy; camilla.mattiuzzi@apss.tn.it

Laboratory medicine is a cornerstone of modern clinical practice, providing essential contributions to screening, diagnosis, prognosis and therapeutic management of human disease [1]. Recent advances in laboratory diagnostics have sparked a transformative revolution by enabling more accurate, rapid, and cost-effective testing methodologies [2]. These innovations span across several domains, including traditional phenotypic assays, molecular diagnostics, point-of-care technologies, digital integration, and artificial intelligence (AI)-driven approaches [3]. This Special Issue therefore aims to showcase the technological and translational progress that meaningfully advances patient care through laboratory medicine, promoting a deeper understanding of how laboratory medicine continues to evolve to meet the dynamic needs of contemporary healthcare. Valuable insights from various investigations are anticipated.

The first article of this Special Issue by Kardjadj et al., based on the multicenter NCT06996301 trial, evaluated the use of the delta cycle threshold value (ΔCt; i.e., the difference between target gene and internal control Ct values) as a semi-quantitative measure of bacterial load in urinary tract infections when paired with multiplex polymerase chain reaction (PCR) panels [4]. Analyzing 1027 paired PCR and quantitative urine culture specimens across six sites, the authors found that PCR positivity was higher than culture positivity, likely due to detection of non-viable DNA or fastidious pathogens. Strong correlations were observed between ΔCt and bacterial load (log10 colony-forming unit (CFU)), with species-specific models indicating that each ΔCt unit change corresponds to a 5.6–8.4-fold change in CFU. A receiver operating characteristic (ROC) analysis showed good discrimination (between 0.78 and 0.84) for predicting high CFU levels but did not enable a perfect match. The study also revealed that the specimen collection method did not significantly affect the ΔCt-to-CFU relationship, but delays in processing reduced CFU recovery. This trial’s conclusions hence support the use of ΔCt as a reproducible, analytically valid semi-quantitative marker for urinary bacterial load, although assay-specific cut points and handling controls must be validated before use in clinical settings.

The second article, published by the same team of authors, refers again to the recent findings from the NCT06996301 clinical trial [5] aiming to explore antimicrobial resistance detection in adults with complicated urinary tract infections. They assessed the performance of rapid, multiplex PCR-based molecular diagnostics for identifying antimicrobial resistance genes, comparing genotypic results with phenotypic antimicrobial susceptibility patterns and exploring the relationship between quantitative PCR Ct values and minimum inhibitory concentrations. High concordance was observed for key resistance determinants, especially bla_CTX-M_ in *E. coli*, for which sensitivity and specificity approached 94% and 99.5%. Ct values showed modest but statistically significant correlations with MICs for selected genes, suggesting that quantitative PCR outputs may not only identify resistance determinants but also provide insights into the magnitude of resistance or detect heteroresistant subpopulations. From a clinical standpoint, PCR-guided therapy markedly reduced the time to effective antimicrobial treatment (20 versus 52 h in culture-guided management) and improved therapeutic success (88.1% vs. 78.1%)

Next is an article by Dossou et al. [6], who evaluated the analytical performance of the i-Tracker chemiluminescent immunoassays (CLIA) on the IDS-iSYS system for monitoring therapeutic drug levels and anti-drug antibodies (ADAs) in patients treated with biologics Adalimumab and Infliximab. These innovative drugs are commonly used to treat autoimmune diseases, where monitoring drug and ADA levels is crucial for optimizing treatment. The i-Tracker assays demonstrated excellent linearity, accuracy and precision within clinically relevant ranges. Compared with a gold-standard electrochemiluminescent immunoassay, the drug assays showed strong concordance and minimal bias. The total anti-Adalimumab assay demonstrated over 85% qualitative agreement, whereas the total anti-Infliximab assay showed a higher ADA detection rate but lower negative agreement with the reference technique. Importantly, the assays detected ADAs even at supratherapeutic drug concentrations. The results of this robust analytical study thus support the clinical use of i-Tracker for therapeutic drug monitoring in patients receiving biologics.

The article by Asim et al. reports the findings of a retrospective study that aimed to assess the clinical relevance of rotational thromboelastometry (ROTEM) in detecting early coagulopathy in trauma patients [7]. Analyzing 1488 adult trauma cases, the authors found that 25.3% of patients exhibited ROTEM abnormalities on admission, especially reduced FIBrinogen TEster with platelet inhibition (FIBTEM) maximum clot firmness (MCF). The results correlated with hypofibrinogenemia and worse clinical outcomes, including increased transfusion requirements and mortality, demonstrating a significant positive association between plasma fibrinogen concentration and FIBTEM MCF. These results underscore the diagnostic utility of ROTEM across a spectrum of injury severities and advocate for broader integration of this test in trauma resuscitation protocols in prospective studies.

Fagnani et al. evaluated the analytical performance of a turbidimetric immunoassay for assessing hemoglobin A1c (HbA1c) [8], a crucial parameter for diagnosis and management of diabetic patients. Compared to a high-performance liquid chromatography (HPLC) gold standard, this automated diagnostic technique showed excellent correlation with minimal bias and high precision across the clinical decision ranges. The assay demonstrated coefficients of variation between 0.68 and 2.4%, indicating strong repeatability and excellent within-laboratory imprecision, thereby minimizing the risk of diagnostic misinterpretations. The results of the study position this immunoturbidimetric assay as a reliable and cost-effective alternative for routine HbA1c measurement, especially suitable for laboratories with limited resources.

In the next article, Kim and colleagues addressed the limitations of the Fibrosis-4 (FIB-4) index in detecting advanced hepatic fibrosis in patients with type 2 diabetes mellitus (T2DM) and metabolic dysfunction-associated steatohepatitis (MASD) [9]. By analyzing liver biopsy data from 1503 patients (34% with T2DM), they developed a modified FIB-4 model called “Diabetes Fibrosis Index”, which incorporates additional parameters such as the aspartate aminotransferase-to-alanine aminotransferase ratio (AST/ALT), albumin, triglycerides and platelets. This model demonstrated modestly improved diagnostic accuracy, with an area under the curve of 0.771 compared to 0.735 for the conventional FIB-4, and a higher negative predictive value across age groups in the diabetic cohort. The non-linear regression approach tailored to the T2DM population could enhance the screening of advanced fibrosis, supporting personalized risk stratification in MASD.

Njoku et al. investigated the use of the C-C Motif Chemokine Receptor 5 (*CCR5*) gene as a molecular biomarker to assess sample adequacy in human papillomavirus (HPV) molecular diagnostics [10]. Using Jurkat cell line samples, the study demonstrated that quantification of *CCR5* using real-time PCR strongly correlated with cell counts obtained from traditional methods such as Thoma chamber and fluorescence-activated cell sorting. This finding validates *CCR5* as a reliable endogenous control for accurately quantifying cellularity in HPV testing, reducing the risk of false-negative results due to insufficient sample quality and improving molecular diagnostic accuracy in cervical cancer screening and HPV-related disease management.

In concert with these emerging technical innovations, Czarnobilska et al. analyzed the role of the Basophil Activation Test (BAT) in diagnosing drug-induced anaphylaxis [11]. The authors studied 150 patients suspected of drug allergies, observing that the BAT had varying sensitivity and specificity depending on the drug tested. For example, the sensitivity and specificity were 40% and 75% for the coronavirus disease 2019 (COVID-19) vaccine, while they were 100% and 75% for polyethylene glycol (PEG) 4000. Positive BAT results were obtained for multiple antibiotic agents, non-steroidal anti-inflammatory drugs (NSAIDs), and local anesthetics. The study finally concluded that BAT could be considered a useful, safer, and cost-effective alternative to in vivo provocation tests, which can confirm allergy and potentially avoid unnecessary drug challenges and associated anaphylactic risks in clinical allergy diagnosis.

Ciubotaru and colleagues reviewed the use of neurodegenerative biomarkers in multiple sclerosis (MS) [12], a complex autoimmune disorder characterized by progressive neurodegeneration that leads to disability. The article outlined recent advances in cerebrospinal fluid and blood biomarkers, including neurofilaments, lipid metabolites, and kynurenine pathway metabolites, which reflect neuroaxonal damage. The authors concluded that integrating these biomarkers into clinical practice could enhance MS diagnosis, disease monitoring, and prognostication, thereby bridging the gap between research discoveries and clinical application.

In their narrative review, Samavarchitehrani et al. explored insulin resistance (IR) indices, specifically the Homeostatic Model Assessment for IR (HOMA-IR) and the triglyceride-glucose (TyG) index, within the context of managing fibromyalgia [13]. Fibromyalgia patients often exhibit metabolic abnormalities, including IR, potentially associated with mitochondrial dysfunction and oxidative stress. Available evidence suggests that these patients have higher IR levels compared to controls, with HOMA-IR correlating with disease severity scores. They concluded that further large-scale research is needed to clarify the causal role of IR in the pathophysiology of fibromyalgia.

Advancing along this compelling trajectory of laboratory innovations, Cancado et al. challenged the longstanding serum ferritin threshold used for diagnosing iron deficiency, currently set at <15 ng/mL by the World Health Organization (WHO) standards [14]. Recognizing that non-anemic iron deficiency may be far more prevalent and underdiagnosed, the authors argue that a higher serum ferritin cutoff of <50 ng/mL may better reflect physiological iron status and correlate with hepcidin regulation. Raising this threshold could improve the diagnosis of iron deficiency, especially for populations at higher risk of this condition (i.e., young women). The article advocates the global revision of serum ferritin diagnostic criteria to enhance the timely detection and treatment of iron deficiency.

In the next article, Favaloro and Pasalic discuss recent breakthrough innovations in hemostasis diagnostics, highlighting the evolution from traditional coagulation tests to advanced assays that integrate non-standard clotting techniques such as chemiluminescence and flow cytometry [15]. The review covers emerging technologies designed to assess hemostatic balance more comprehensively, including tests that evaluate both primary and secondary hemostasis. The authors also explore future directions, including AI applications for diagnostics and development of global assays to optimize the monitoring of evolving therapies aimed at rebalancing hemostasis.

An insightful analysis is then presented by Tiwari et al. [16], who reviewed the emerging role of natriuretic peptides (NPs), notably B-type natriuretic peptide (BNP) and N-terminal proBNP (NT-proBNP), in diabetes care. Given the high risk of heart failure among individuals with diabetes, elevated NP levels may offer valuable prognostic information regarding cardiac structure and function. The review highlighted the utility of NP testing in risk stratification and therapeutic guidance in T2DM while discussing analytic challenges and the potential of NP-guided therapy to improve cardiovascular outcomes. The authors suggested future research directions to optimize the use of NPs in this high-risk population.

The final article, which concludes this Special Issue, contains an overview by Lippi et al. on the shifting paradigm in laboratory medicine towards patient self-testing with portable and wearable devices [17]. This decentralized testing model contrasts with earlier trends of centralization, aiming to bring diagnostic testing closer to patients for enhanced convenience and monitoring. The review discusses the advantages and limitations of various home, portable, and implantable devices, emphasizing their potential to coexist with traditional laboratory services depending on patient needs and clinical contexts. The article concludes with some reflections on the transformative impact of technology on diagnostic pathways and patient-centered care models.

Collectively, the articles featured in this Special Issue on advances in the laboratory diagnosis illustrate the accelerating pace at which technological innovation is redefining the scope, precision, and clinical impact of laboratory medicine. Across diverse domains, from immunoassay optimization, and molecular diagnostics to biomarker discovery, risk stratification tools, and decentralized testing models, emerging technologies are reshaping the accuracy, speed, and interpretability of diagnostic information. These advances are not merely incremental as they generate a profound impact on diagnostic pathways, enabling earlier and more targeted interventions and supporting increasingly personalized approaches to patient care [18].

Several unifying themes are evident, most notably the growing integration of molecular, digital, and analytical methodologies. This convergence is strengthening the association between laboratory data and clinical decision-making, supporting more precise and effective patient management. These developments are fully consistent with the principles of value-based laboratory medicine, which prioritize not only advances in therapeutic drug monitoring, rapid detection of pathogens and antimicrobial resistance, and biomarker-guided disease management, but also the enhancement of clinical utility and overall healthcare value through more efficient, impactful, and sustainable diagnostic testing [19,20]. The adoption of rapid and high-resolution diagnostic platforms is substantially increasing throughput, reducing turnaround time and ultimately improving clinical outcomes, especially in time-dependent conditions such as trauma and infectious and metabolic disease. The expanding role of AI, automated analytics, and patient-operated testing devices is ultimately fostering a more integrated and patient-centered diagnostic ecosystem that extends beyond the traditional laboratory environment [21].

As healthcare systems face rising pressures from a constantly increasing burden of chronic diseases, antimicrobial resistance, and increasing expectations for (more personalized) care, the innovations highlighted in this Issue underscore the indispensable role of laboratory medicine in addressing these important challenges. Continued investments in technology, assay development, validation and standardization, along with translational research, will be essential to fully realize the potential of next-generation laboratory diagnostics. The future of laboratory medicine will be defined by its ability to transform sophisticated scientific advances into accessible, actionable tools that elevate patient care, promote stewardship, and support equitable healthcare delivery worldwide [22].

## Data Availability

No new data were created or analyzed in this study. Data sharing is not applicable to this article.

## Abbreviations

The following abbreviations are used in this manuscript:

AI	Artificial Intelligence
PCR	Polymerase chain reaction
Ct	Cycle threshold
CLIA	Chemiluminescent immunoassays
ADAs	Anti-drug antibodies
ROTEM	Rotational thromboelastometry
FIBTEM	FIBrinogen TEster with platelet inhibition
MCF	Maximum clot firmness
HbA1c	Hemoglobin A1c
HPLC	High-performance liquid chromatography
MASD	Metabolic dysfunction-associated steatohepatitis
FIB-4	Fibrosis-4
T2DM	Type 2 diabetes mellitus
CCR5	C-C Motif Chemokine Receptor 5
HPV	Human papillomavirus
BAT	Basophil Activation Test
COVID-19	Coronavirus Disease 2019
PEG	Polyethylene glycol
NSAIDs	Non-steroidal anti-inflammatory drugs
MS	Multiple sclerosis
IR	Insulin resistance
HOMA-IR	Homeostasis Model Assessment of IR
TyG	Triglyceride-glucose index
WHO	World Health Organization
NPs	Natriuretic peptides
BNP	B-type natriuretic peptide
NT-proBNP	N-terminal pro-B-type natriuretic peptide
